# New Lipid Mediators in Retinal Angiogenesis and Retinopathy

**DOI:** 10.3389/fphar.2019.00739

**Published:** 2019-07-05

**Authors:** Ingrid Fleming

**Affiliations:** ^1^Institute for Vascular Signalling, Centre for Molecular Medicine, Goethe-University, Frankfurt, Germany; ^2^German Centre for Cardiovascular Research (DZHK) partner site RheinMain, Frankfurt, Germany

**Keywords:** 19,20-dihydroxydocosapentaenoic acid, cytochrome P450, diabetic retinopathy, epoxyeicosatrienoic acid, soluble epoxide hydrolase

## Abstract

Retinal diseases associated with vascular destabilization and the inappropriate proliferation of retinal endothelial cells have major consequences on the retinal vascular network. In extreme cases, the development of hypoxia, the upregulation of growth factors, and the hyper-proliferation of unstable capillaries can result in bleeding and vision loss. While anti-vascular endothelial growth factor therapy and laser retinal photocoagulation can be used to treat the symptoms of late stage disease, there is currently no treatment available that can prevent disease progression. Cytochrome P450 enzymes metabolize endogenous substrates (polyunsaturated fatty acids) to bioactive fatty acid epoxides that demonstrate biological activity with generally protective/anti-inflammatory and insulin-sensitizing effects. These epoxides are further metabolized by the soluble epoxide hydrolase (sEH) to fatty acid diols, high concentrations of which have vascular destabilizing effects. Recent studies have identified increased sEH expression and activity and the subsequent generation of the docosahexaenoic acid-derived diol; 19,20-dihydroxydocosapentaenoic acid, as playing a major role in the development of diabetic retinopathy. This review summarizes current understanding of the roles of cytochrome P450 enzyme and sEH–derived PUFA mediators in retinal disease.

## The CYP-sEH Pathway and its Biological Actions

Cytochrome P450 (CYP) enzymes are membrane-bound, heme-containing oxidases that are part of a multi-enzyme complex that includes cytochrome P450 reductase and cytochrome b5; for review see Spector and Kim (2015) and Hu et al. (2018). CYP enzymes are responsible for the metabolism of numerous pharmaceutical compounds, but they also utilize endogenous compounds as substrates, including cholesterol and polyunsaturated fatty acids (PUFAs). CYP enzymes are most highly expressed in the liver but are also present in the kidney, skeletal muscle, adipose tissue, pancreas, and vasculature and metabolize PUFAs to either epoxides or ω-hydroxides.

**Figure 1:**
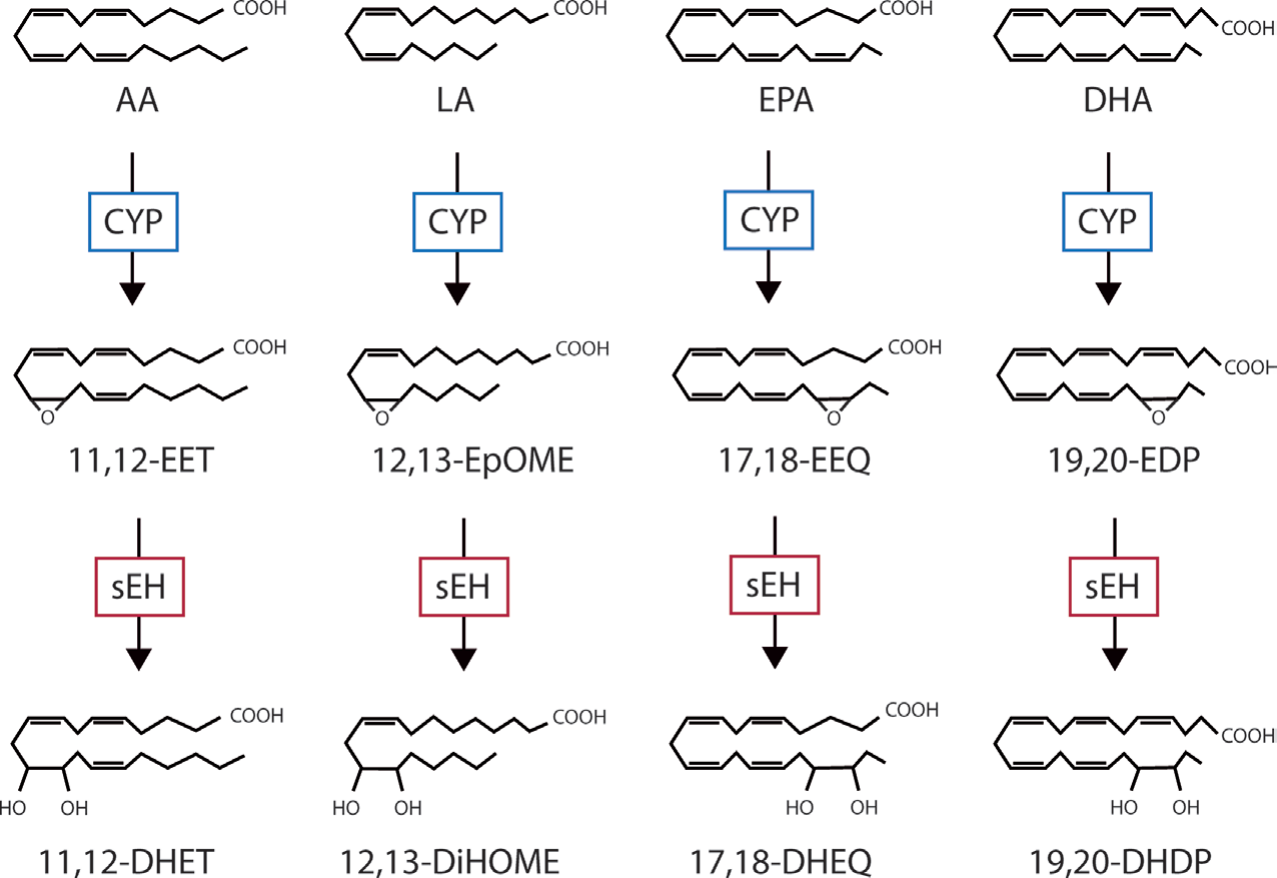
The cytochrome P450 (CYP)/soluble epoxide hydrolase (sEH) axis and polyunsaturated fatty acid (PUFA) metabolism. Examples of the mediators generated as by CYP- and sEH-dependent metabolism of the ω-6 PUFAs; arachidonic acid (AA) and linoleic acid (LA), as well as the ω-3 PUFAs; eicosapentaenoic acid (EPA) and docosahexaenoic acid (DHA). EET, epoxyeicosatrienoic acid; DHET, dihydroxyeicosatrienoic acid; EEQ, epoxyeicosatetraenoic acid; DHEQ, dihydroxyeicosatetraenoic acid; EpOME, epoxyoctadecamonoenic acid; DiHOME, dihydroxyoctadecenoic acid; EDP, epoxydocosapentaenoic acid; DHDP, dihydroxydocosapentaenoic acid.

The best-known PUFA is arachidonic acid, a 20-carbon ω-6 PUFA that in humans is derived from linoleic acid taken up from the diet. Arachidonic acid can be metabolized by a number of different enzymes including cyclooxygenases, lipoxygenases, and CYP enzymes, each generating different products with distinct chemical properties and biological actions. In the vascular system, smooth muscle cells are often linked with the CYP4A-mediated metabolism of arachidonic acid to hydroxyeicosatetraenoic acids (HETEs)—which have vasoconstrictor properties. Endothelial cells, on the other hand, are reported to generate mostly epoxyeicosatrienoic acids (EETs) which are linked with vasodilatation and decreased blood pressure; for review see Fleming (2014). However, CYP enzymes can demonstrate a mixed function and can generate both metabolites with the ratio of EETs to 20-HETE varying between the specific CYP isoforms. It is not only the expression profile of the PUFA metabolizing enzymes that determines the spectrum of metabolites generated in a given cell type or tissue but also its PUFA makeup. For example, while ω-6 PUFAs (e.g. arachidonic and linoleic acid) dominate in the liver and vascular cells in the systemic circulation, the situation is very different in the brain and retina where levels of the ω-3 PUFAs docosahexaenoic acid (DHA) and eicosapentaenoic acid (EPA) are much higher than those of arachidonic acid (Arterburn et al., 2006; Hård et al., 2013). This is relevant as CYP–derived metabolites of arachidonic acid and linoleic acid have generally been attributed to pro-inflammatory actions (Viswanathan et al., 2003; Saraswathi et al., 2004), while the metabolism of ω-3 PUFA’s results in the generation of metabolites generally attributed anti-inflammatory effects (Morin et al., 2010; López-Vicario et al., 2015; Schunck et al., 2018). The biological activity of the PUFA epoxides is regulated by their hydrolysis to diols by the epoxide hydrolases that, for example, generate dihydroxyeicosatrienoic acids (DHETs) from the EETs (**Figure 1**); although 5,6–EET seems to be a preferred substrate for cyclooxygenase enzymes. The best studied epoxide hydrolase is the soluble epoxide hydrolase (sEH; gene name *Ephx2*); reviewed in Morisseau and Hammock (2005)and Hiesinger et al. (2019), but there are three other members of the protein family that include the microsomal epoxide hydrolase (gene name *Ephx1*) as well as EPHX3 and EPHX4 (Decker et al., 2012). While the microsomal epoxide hydrolase has also been implicated in the regulation of epoxide diol levels in some situations (Edin et al., 2018) and EPHX4 has yet to be studied in detail, *Ephx3* ^−/−^ mice do not demonstrate clear alterations in epoxide:diol ratios even though the enzyme was reported to catalyze the hydrolysis of 11,12-EET and the linoleic acid epoxide 9,10-epoxyoctadecamonoenoic acid (EpOME) *in vitro* (Hoopes et al., 2017). The hydrolysis of the PUFA epoxides was initially thought to represent an inactivation process as some diols possess biological activity only at high concentrations (Fang et al., 2006; Huang et al., 2017). More recent studies have however identified physiological roles for diols of linoleic acid in the regulation of liver (Mangels et al., 2016), adipose tissue (Lynes et al., 2017), and skeletal muscle metabolism (Stanford et al., 2018), pain (Zimmer et al., 2018), as well as stem cell proliferation and mobilization (Frömel et al., 2012).

While there have been significant advances in knowledge regarding the role of CYP- and sEH-derived PUFA metabolites in cardiovascular disease; for review see Gross and Nithipatikom (2009), renal disease; for review see Imig (2018), and even metabolic syndrome and diabetes; for review see Huang et al. (2016), comparatively little is known about the impact of this pathway in the eye.

## Mechanism(s) of Action

### Epoxides

**Figure 2:**
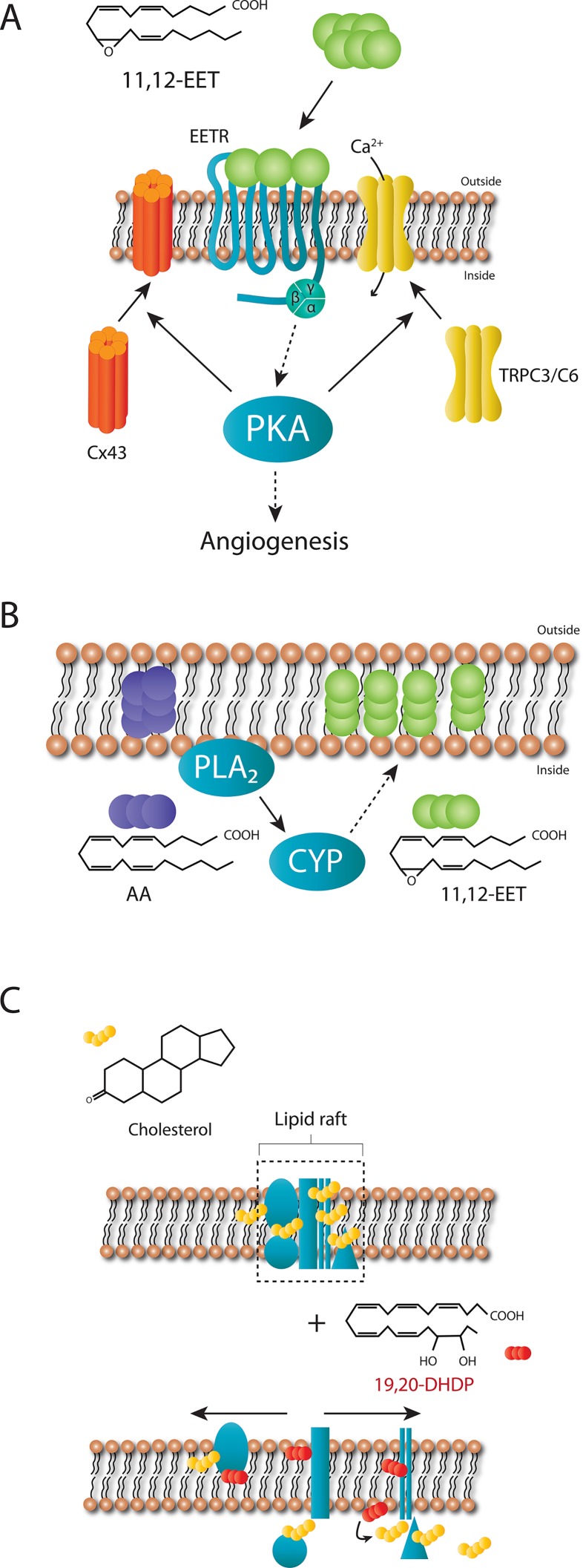
Proposed mechanism of actions of 11,12-EET and 19,20-DHDP. **(A)** Extracellular 11,12-EET binding to a specific, high affinity receptor coupled to Gαs on cell membranes can account for the dependence of 11,12-EET-induced connexin (Cx) 43 and TRPC3/C6 translocation as well as angiogenesis on protein kinase A (PKA). **(B)** EETs generated from arachidonic acid after its liberation from the plasma membrane can also be incorporated into phospholipids to act “intracellular stores” of reactive epoxides. **(C)** Cholesterol-rich micro domains of the plasma membrane (lipid rafts) contain complexes of proteins, several of which have been identified as cholesterol binding proteins. 19,20-DHDP is able to displace cholesterol and cholesterol-binding proteins from these micro domains to disrupt lipid rafts and as a consequence the function(s) of such complexes, one example being the γ-secretase.

The most studied PUFA epoxides are those derived from arachidonic acid. CYP enzymes can metabolize arachidonic acid to generate four EET regioisomers, i.e. 5,6-EET, 8,9-EET, 11,12-EET, and 14,15-EET. Each of these EETs can occur in an R,S- or S,R-enantiomeric configuration, which potentially exert different effects. The EET’s (particularly 11,12- and 14,15-EET) have been implicated in the acute nitric oxide and prostacyclin-independent regulation of vascular tone (Campbell et al., 1996; Fisslthaler et al., 1999) as well as with longer term processes such as angiogenesis (Medhora et al., 2003; Michaelis et al., 2003). The mechanism of action has yet to be definitively clarified but evidence indicates that a Gαs-coupled membrane receptor exists (**Figure 2A**), at least for 11,12-EET (Inceoglu et al., 2007; Campbell and Fleming, 2010). For example, 11,12-EET increases GTPγ^35^S binding to Gs, but not Gi proteins (Node et al., 2001), and the small interfering RNA-induced downregulation of Gas (but not Gq/11) abrogated the 11,12-EET-induced translocation of connexin subunits of gap junction plaques (Popp et al., 2002), and transient receptor potential (TRP) channels as well as the initiation of angiogenesis (Ding et al., 2014). Added to this many of the effects of 11,12-EET are dependent on the activation of protein kinase (PK) A (Wong et al., 2000; Fukao et al., 2001; Popp et al., 2002; Fleming et al., 2007), and EET binding sites have been described on the surface of cells known to respond to the epoxides (Yang et al., 2008; Chen et al., 2009; Pfister et al., 2010; Chen et al., 2011). Cellular responses to EETs are also highly dependent on regioisomer applied (i.e. 5,6- versus 8,9-, 11,12-, or 14,15-EET) as well as on the stereoisomer. Indeed, when compared with 11(S),12(R)-EET, 11(R),12(S)-EET is a more potent activator of K_Ca_ channels in renal arteries (Zou et al., 1996), and rat airways (Pascual et al., 1998) as well as endothelial cell TRP channel translocation (Ding et al., 2014). The existence of at least one EET receptor can also explain the effectiveness of the so-called EET agonists and antagonists that have been developed over the last 18 years (Gauthier et al., 2002; Falck et al., 2003; Yang et al., 2008). While it seems likely that the as yet unidentified 11,12-EET receptor is a selective high affinity receptor for one specific PUFA epoxide, two low affinity receptors have been linked with EET-induced cellular responses. The first is GPR40, which is also known as free fatty acid receptor 1, and has been linked with Alzheimer’s disease and the dementia associated with type 2 diabetes (Chen et al., 2019). GPR40 overexpression potentiated the effects of 11,12- and 14,15-EET on the proliferation of cell lines *via* the aforementioned crosstalk with the epidermal growth factor receptor (Ma et al., 2015). Also in GPR40 overexpressing HEK cells, 11,12-EET and to a lesser extent 8,9- and 5,6-EET, as well as 11,12- and 14,15-DHET were able to elicit a Ca^2+^ response that was sensitive to a GPR40 antagonist (Park et al., 2018). Although GPR40 is expressed by endothelial cells and smooth muscle cells, and the 11,12-EET-induced kinase activation as well as connexin phosphorylation and cyclooxygenase expression in cultured endothelial cells were sensitive to GPR40 antagonism, the 11,12-EET-induced relaxation of coronary arteries was not (Park et al., 2018). The second potential low affinity receptor for EETs, identified using a bioinformatic approach, is GPR132 (Lahvic et al., 2018). The link between this receptor and 11,12-EET could be demonstrated *in vivo* as GPR132 knockdown prevented EET-induced hematopoiesis in zebrafish, and bone marrow cells from GPR132 knockout mice showed decreased long-term engraftment capability. Like GPR40, GPR132 also responds to hydroxy-fatty acids (Lahvic et al., 2018). CYP-derived epoxides and diols may also possess intracellular receptors. For example, EETs can activate peroxisome proliferator-activated receptors (PPARs) (Fang et al., 2006), which may account for some of the EET-induced effects in hepatic and endothelial inflammation, as well as adipocyte differentiation (Kim et al., 2010; Morin et al., 2010; Barbosa-da-Silva et al., 2015; Li et al., 2015; Huang et al., 2017; Veiga et al., 2017)..

PUFA-derived epoxides and diols can also initiate cell signaling *via* membrane receptor-independent mechanisms as they can incorporate into membrane phospholipids to alter the membrane lipid composition and fluidity (Kitson et al., 2012). For example, the fish oils EPA and DHA can be incorporated into phospholipids and infiltrate lipid rafts as well as alter the composition of non-raft domains, with DHA incorporating more readily than EPA (Williams et al., 2012). This may well be a characteristic of diols as the EET-derived DHETs can also be incorporated into phosphatidylcholine and phosphatidylinositol (Capdevila et al., 1987; Karara et al., 1991; VanRollins et al., 1996; Nakamura et al., 1997; Fang et al., 2003). The conjugated phospholipids described in the latter reports have yet to been characterized in detail using more modern mass spectrometry–based methods and it remains to be determined whether or not the modified phospholipids are effectors in themselves or act as “intracellular stores” of reactive epoxides and diols. For example, the incorporation of EETs into a phospholipid pool (**Figure 2B**), was suggested to represent a storage mechanism as it could be catalyzed by acyl coenzyme synthase in endothelial cells (Weintraub et al., 1997), while a similar protein kinase C (PKC)-mediated phenomenon was described in astroglia (Shivachar et al., 1995).

### Diols

Relatively little is known about the biological actions of the PUFA diols, largely because the sEH-dependent metabolism of the epoxides was initially assumed to inactivate epoxide-initiated signaling. Perhaps the first diols attributed a biological action were derived from linoleic acid i.e., 9,10- and 12,13-dihydroxy-9Z-octadecenoic acid (DiHOME). The identification of a link between these mediators and adult respiratory stress syndrome (ARDS) is an interesting one and stemmed from the observation that circulating levels of linoleic acid were decreased in ARDS patients (Quinlan et al., 1996; Kumar et al., 2000). Initially the metabolism of linoleic acid to its epoxides was linked with toxicity, particularly in patients with severe burns that went on to develop respiratory problems (Fukushima et al., 1988; Ozawa et al., 1991; Kosaka et al., 1994). Indeed, 9,10-epoxyoctadecenoic acid (EPOME) is also referred to as leukotoxin. However, it turned out that these epoxides only elicited cytotoxicity in cells expressing the sEH, meaning that the truly toxic mediators were the diols or DiHOMEs (Moghaddam et al., 1997). Indeed, higher levels of 9,10- and 12.13-DiHOME were detected in patients with acute respiratory distress (Moghaddam et al., 1997). A direct comparison of the actions of leukotoxin and leukotoxin diol (9,10-DiHOME) in mice *in vivo*, revealed that only the animals that received the diol developed massive alveolar edema and hemorrhage with interstitial edema around blood vessels and died of ARDS-like respiratory distress (Zheng et al., 2001). Importantly, inhibiting the sEH decreased the mortality induced by the epoxide but not by the diol. Although these observations implied that sEH inhibitors could be used to treat ARDS, the simpler approach was to replace the linoleic acid in the parenteral nutrition with fish oils (ω-3 PUFAs); an intervention that resulted in marked benefits in gas exchange, ventilation requirement, and mortality (Calder, 2010). The CYP/sEH-dependent generation of leukotoxin-diol (9,10-DiHOME) in goblet cells has also been recently implicated in a form of chronic obstructive pulmonary disease most frequently experiences by females (Balgoma et al., 2016).

While very high concentrations of linoleic acid-derived diols in critically ill patients are clearly detrimental, the more general use of sEH inhibitors in different models as well as the global and tissue-specific deletion of the sEH has helped to identify biological actions of specific diols under physiological conditions (Fleming, 2014). For example, low concentrations of 12,13-DiHOME have been reported to inhibit the respiratory burst in neutrophils (Thompson and Hammock, 2007). In the zebrafish as well as in the mouse, sEH derived diols have been linked with the proliferation and mobilization of hematopoietic cells. The first study of the role of the sEH in the zebrafish was aimed at assessing its role in angiogenesis. However, rather than detecting an effect on sprouting angiogenesis, both the inhibition and the knockdown of the sEH initiated a defect in the caudal vein plexus and decreased the numbers of lmo2/cmyb-positive progenitor cells therein (Frömel et al., 2012). The latter is a complex vessel network that originates from the caudal vein 24–48 h post fertilization and serves as a transient hematopoietic tissue (Murayama et al., 2006). MS-based profiling of zebrafish embryos identified 12,13-DiHOME and 11,12-DHET as the sEH products most altered by enzyme inhibition and both of these diols were able to rescue the hematopoietic cell phenotype (Frömel et al., 2012). Mechanistically, the action of the diols in the zebrafish embryos was linked to Wnt signaling (Frömel et al., 2012). These findings could also be transferred to mice and the sEH was found to be highly expressed in bone marrow cells particularly in bone marrow–derived hematopoietic progenitor cells i.e. lineage negative (Lin-) cKit positive (cKit+) cells. The latter were able to generate 9,10- and 11,12-EpOME/DiHOME as well as 11,12- and 14,15-EET/DHET (Frömel et al., 2012). Functionally, bone marrow cells from sEH^−/−^ mice formed significantly fewer colonies than cells from wild-type mice, a response that could be rescued by adding either 11,12-DHET or 12,13-DiHOME. The Lin-Sca-1+cKit+ cells affected by sEH inhibition and deletion go on to give rise to neutrophils and monocytes, which contribute to angiogenesis. Therefore, it is not surprising that the vascularization of an acellular matrix was more successful in the wild-type than in the sEH^−/−^ mice. More importantly, in a model of hindlimb ischemia induced by the ligation of the femoral artery the recovery of normal blood flow was markedly delayed in sEH deficient animals. The latter effect was dependent on bone marrow derived cells as it could be rescued by bone marrow transplantation with wild-type bone marrow. Consistent with the effects of 12,13-DiHOME on progenitor cells, the infusion of this diol also accelerated the recovery of blood flow following ischemia in the sEH^−/−^ mice, making them similar to the wild-type (Frömel et al., 2012). Since these reports, 12,13,DiHOME has been identified as a lipokine, or circulating lipid mediator, released from brown adipose tissue following cold exposure (Lynes et al., 2017). Mechanistically, 12,13-DiHOME elicited the translocation of the fatty acid transporters FATP1 and CD36 to the cell membrane to increase fatty acid uptake. Similarly, moderate-intensity exercise was reported to increase circulating 12,13-DiHOME levels in humans as well as in mice. The lipokine was supposedly derived from brown adipose tissue as its deletion prevented 12,13-DiHOME generation (Stanford et al., 2018). These observations led to the proposal that, while “cold causes the release of 12,13-DiHOME from brown adipose tissue to function in an autocrine manner to provide fuel for brown adipose tissue, exercise causes the release of 12,13-DiHOME from brown adipose tissue to function in an endocrine manner, resulting in stimulation of fatty acids into the working skeletal muscle” (Stanford et al., 2018). The latter studies did not determine the consequences of altered sEH expression on 12,13-DiHOME or fatty acid metabolism. It will be interesting to determine whether the attenuated exercise capacity described for sEH^−/−^ mice (Keserü et al., 2010), is in any way related to decreased circulating 12,13-DiHOME and altered fatty acid uptake into skeletal muscle, rather than the initially proposed changes in the pulmonary vasculature.

A more recently described diol is the DHA-derived 19,20-DHDP that is required for the optimal development of the vascular plexus in the mouse retina. To do this 19,20-DHDP inhibits the γ-secretase complex by altering the subcellular localization of the presenilin 1 (PS-1) within the plasma membrane and dislocating it from lipid rafts (Hu et al., 2014). Mechanistically, DHA and 19,20-DHDP are thought to exert their effects independently of a receptor by means of insertion into the lipid bilayer (**Figure 2C**). This phenomenon is also linked to the redistribution of membrane cholesterol and cholesterol-binding proteins from lipid raft to non-lipid raft fractions of the membrane (Huster et al., 1998; Wassall and Stillwell, 2009). The sensitivity of PS-1 to 19,20-DHDP is explained by the fact that it is a cholesterol binding protein (Hulce et al., 2013). When the amount of 19,20-DHDP generated is low, it appears that it plays a central role in the regulation of Notch signaling (Hu et al., 2014). However, when sEH expression is elevated and much more 19,20-DHDP is generated its actions seem to tip toward negative effects. The molecular mechanisms, however, seem to be the same i.e. the interaction with cholesterol and PS-1 binding proteins. Indeed, PS-1 is not only part of the γ-secretase complex and is also localized to tight junctions where it binds to and stabilizes VE-cadherin (Cai et al., 2011) as well as N-cadherin (Georgakopoulos et al., 1999; Baki et al., 2001; Serban et al., 2005), and thus endothelial cell-endothelial cell as well as endothelial cell-pericyte junctions. Given this information, it is perhaps not surprising that high concentrations of 19,20-DHDP in the retina are able to dissolve the association of the proteins and thus breakdown the blood retinal barrier (Hu et al., 2017a), one of the characteristic stages in diabetic retinopathy. It remains to be determined whether or not other PS-1 dependent effects such as γ-secretase dependent and pro-apoptotic effects in mitochondria (Xu et al., 2002; Zeng et al., 2015), can also be affected by 19,20-DHDP.

## CYP-Derived PUFA Mediators and Angiogenesis

Angiogenesis is a tightly regulated and organized process and although numerous studies have addressed the role of specific growth factors and proteins at the different stages of vascular development (Potente et al., 2011), much less is known about the role of PUFA-derived lipid mediators. However, the importance of the CYP-sEH pathway in physiological and pathophysiological angiogenesis has become somewhat clearer since the development of global and tissue selective sEH^−/−^ mice (Sinal et al., 2000; Hu et al., 2014), as well as Cyp2c44^−/−^ mice (Pozzi et al., 2010; Hu et al., 2017b), and humanized mouse models i.e. mice overexpressing specific CYP enzymes, usually in endothelial cells (Panigrahy et al., 2012; Shao et al., 2014).

In endothelial cells, the CYP enzymes were initially linked with the nitric oxide- and prostacyclin–independent vasodilatation of small arteries that was later attributed to the endothelium–derived hyperpolarizing factor or EDHF; for review see Michaelis and Fleming (2006). However, it was not long before both the EET’s and “authentic EDHF” were found to initiate cell signaling and activate a series of different kinases including the extracellular regulated kinases and AKT (Fleming et al., 2001a; Potente et al., 2002; Potente et al., 2003). Even though CYP enzymes are expressed in native endothelial cells the initial link between CYP-derived EETs and angiogenesis was made in a culture system in which astrocytes generated the epoxides that promoted both proliferation and tube formation in endothelial cells (Munzenmaier and Harder, 2000; Zhang and Harder, 2002). The fact that an exogenous source of these lipids was required for such experiments can probably be attributed to the lability of CYP enzyme expression in cultured cells. Indeed, the expression of CYP enzyme protein and mRNA decreases so markedly over the first 48 h of culture than in most cell types the enzymes rapidly becomes almost impossible to detect. However, the restoration of CYP expression to cultured endothelial cells using various overexpression techniques (usually adenoviral) and/or the addition of EET regioisomers can elicit angiogenesis (Medhora et al., 2003; Michaelis et al., 2003). Similarly, the exogenous application of EETs to the chick chorioallantoic membrane increased capillary number and induced their reorientation (Michaelis et al., 2003) as well as the vascularization of Matrigel plugs implanted into wild-type mice (Medhora et al., 2003; Webler et al., 2008b). Also, in rats overexpressing the human CYP2C11 and 2J2 enzymes hindlimb ischemia induced a higher muscle capillary density than in wild-type animals (Wang et al., 2005).

Hypoxia is a well-studied angiogenic stimulus, largely because of its effects on hypoxia-inducible factor (HIF)-1α, which then regulates the expression of growth factors including vascular endothelial growth factor (VEGF). However, hypoxia also affects the CYP-sEH pathway in a manner that is biased toward the accumulation of PUFA epoxides. Indeed, the expression of many CYP enzymes is upregulated by hypoxia, and low oxygen tensions increase CYP2C expression and EET production in retinal endothelial cells (Michaelis et al., 2008), while the sEH expression is attenuated in hypoxic conditions *in vitro* and *in vivo* (Keserü et al., 2010). There also seem to be close links with CYP activation and angiogenic growth factor signaling as 11,12 and 14,15-EET are able to initiate crosstalk with the epidermal growth factor receptor (Chen et al., 1998; Chen et al., 2002; Michaelis et al., 2003). Moreover, EETs seem to be a *bona fide* part of the VEGF signaling cascade as a so-called “EET antagonist” was found to prevent VEGF-induced endothelial cell tube formation factor (Webler et al., 2008a; Yang et al., 2009). Interestingly, not only does VEGF increase CYP expression and epoxide generation (Webler et al., 2008a), but CYP enzyme activation increases VEGF expression. 11,12-EET has been proposed to regulate the expression of VEGF by stabilizing HIF-1α (Suzuki et al., 2008; Batchu et al., 2012), the effects of 14,15-EET on VEGF expression, on the other hand, have been attributed to STAT-3 (Cheranov et al., 2008). Additional PUFA-derived mediators i.e. 12-hydroxyeicosatrienoic acid and 20-HETE (Cheng et al., 2014) have been proposed to account for the effects of CYP4B1 (Seta et al., 2007) and CYP4F2 (Cheng et al., 2014), respectively, on VEGF expression. Also VEGF signaling may be potentiated by EETs, as at least 11,12-EET is able to elicit the membrane translocation of the TRPC3 and C6 channels (Fleming et al., 2007) that have also been implicated in VEGF-induced angiogenesis (Hamdollah Zadeh et al., 2008; Andrikopoulos et al., 2017). Epoxides derived from PUFAs other than arachidonic acid can also promote angiogenesis, one example that is relevant to the retina is the 19,20-epoxydocosapentaenoic acid (EDP) derived from DHA (Gong et al., 2016a).

**Figure 3:**
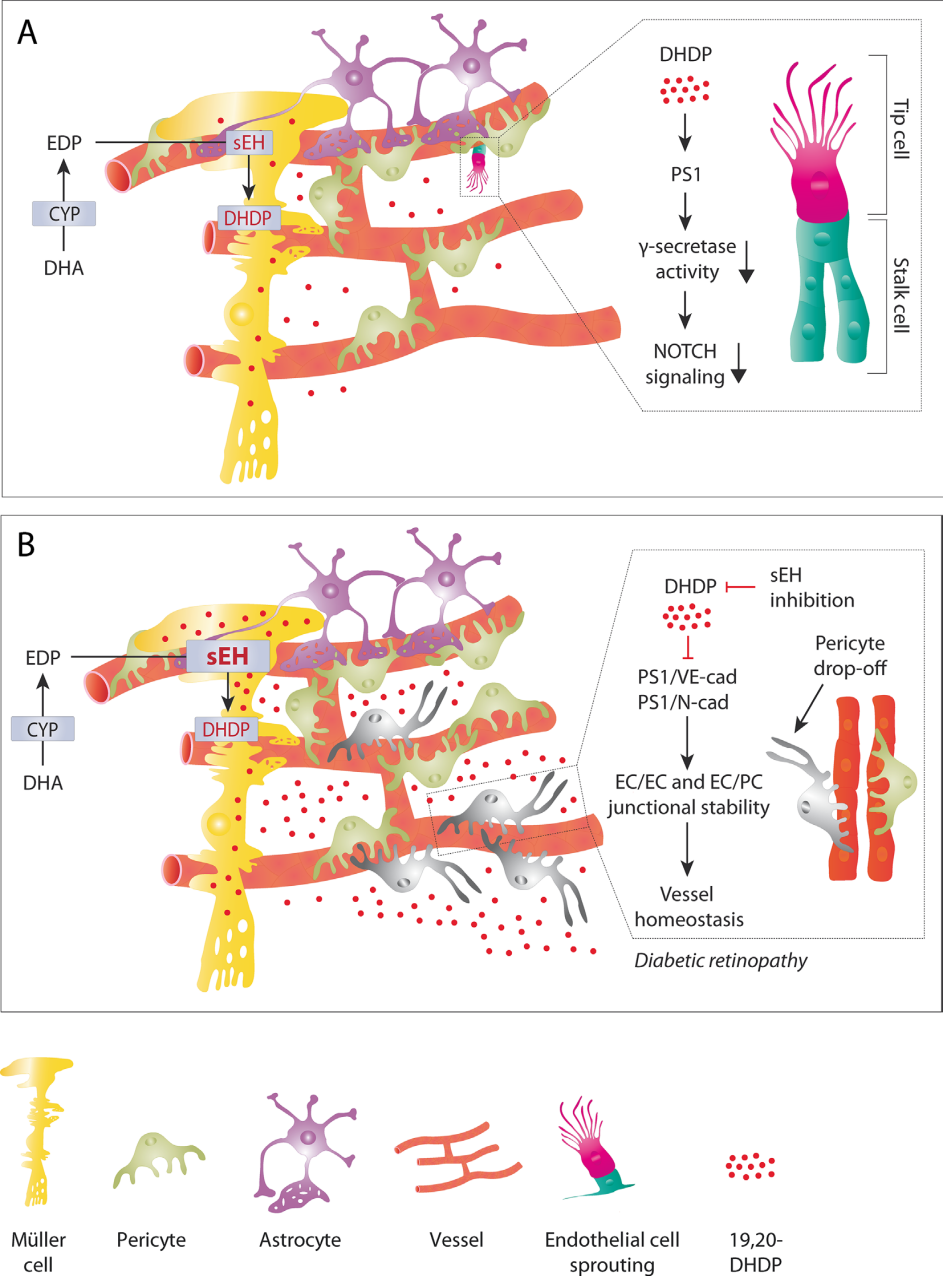
The role of 19,20-DHDP in physiological retinal angiogenesis and retinopathy. **(A)** Physiological (sprouting) angiogenesis. In the retina, the end feet of Müller glial cells are in close contact with the developing vasculature. This means that the 19,20-DHDP generated by Müller cells (and to a lesser extent by astrocytes) can affect the endothelial cell phenotype. By targeting PS1 and inhibiting the γ-secretase 19,20-DHDP interferes with Notch signaling and thus the tip versus stalk cell specification of retinal endothelial cells at the angiogenic front. **(B)** Diabetic retinopathy. Diabetes increases the expression of the sEH in Müller cells, which results in a marked increase in 19,20-DHDP. The high concentrations of 19,20-DHDP target cholesterol binding proteins such as PS1, VE-cadherin, and N-cadherin to dissolve endothelial cell-pericyte contacts. This contributes to the loss of mural cells or pericyte “drop-off.” The simultaneous disruption of endothelial cell-endothelial cell junctions results in increased vascular permeability and loss of barrier function. sEH inhibition effectively prevents the generation of 19,20-DHDP and prevents the development of non-proliferative diabetic retinopathy in a mouse model of type 1 diabetes.

While early *in vitro* studies demonstrated the angiogenic potential of CYP-derived PUFA metabolites such studies could not address the importance of endogenously-generated PUFA mediators. It was also difficult confirm these observations using knockout models as there are major differences in CYP enzyme isoform expression between species and knocking down one specific CYP enzyme frequently results in the upregulation of another that can functionally compensate for it (Holla et al., 2001; Nakagawa et al., 2006). However, clear defects in angiogenesis have been reported in mice lacking Cyp1b1 (Tang et al., 2009; Palenski et al., 2013; Ziegler et al., 2016) and Cyp2c44 (Pozzi et al., 2010; Hu et al., 2017b) as well as mice lacking the sEH (Hu et al., 2014). As noted in the section Diols, one link between the sEH and angiogenesis can be accounted for by the ability of 19,20-DHDP to inhibit the γ-secretase by targeting the membrane localization of PS1. The importance of the γ-secretase lies in its role in the Notch signaling pathway, as it is required to cleave the Notch intracellular domain (NICD) from the Notch receptor proteins, which then translocates to the nucleus to regulate the expression of specific target genes (Lobov and Mikhailova, 2018; Chen et al., 2019). The Notch pathway is a cell-cell signaling cascade where the tip cell (highly migratory cells that sense the VEGF gradient) presents the membrane bound agonist and the stalk cell (highly proliferative cells that for the lumenized vessel) expresses the receptor. The tip cell extends long filopodia that express the VEGF receptor 2 and thus can sense the VEGF gradient, this results in an increase in the Notch agonist delta-like 4 on the tip cell membrane. Activation of the receptor in the adjacent stalk cell elicits the release of the NICD to induce lateral inhibition which basically means that the tip cell induces the stalk cell phenotype in its nearest neighbors (Mack and Iruela-Arispe, 2018). While the VEGF that drives angiogenesis is thought to come from monocytes and astrocytes, the 19,20-DHDP that regulates angiogenesis in the retina comes from Müller glia cells, and to a lesser extent astrocytes (**Figure 3A**). Notch inhibition and activation result in well characterized vascular defects (Lobov and Mikhailova, 2018), but Notch activation mirrors the phenotype induced by sEH deletion and inhibition, which is consistent with the loss of the γ-secretase inhibitor, 19,20-DHDP. In mature vessels, Notch signaling is required for the maintenance of junctional integrity and quiescence as well as arterial fate (Mack and Iruela-Arispe, 2018). Although the role of 19,20-DHDP has not been assessed in detail, high concentrations of DHDP do result in the destabilization of endothelial cell-endothelial cell junctions as well as endothelial cell-pericyte junctions (Hu et al., 2017a).

## Retinal Angiogenesis

One of the first main questions that arose after the realization that interfering with the pathway affected physiological angiogenesis in the mouse eye was the identification of the cell type(s) that expressed the epoxide and diol generating CYP and sEH enzymes. Also the identity of the epoxide and diol species responsible for the effects reported were of interest, especially given the marked differences in the PUFA profiles found in vascular endothelial cells in the systemic circulation and in the retina.

### Endothelial Cells

While in the rest of the body, the CYP enzymes that can generate angiogenic PUFA epoxides have been detected in endothelial cells, this does not seems to be the case in the retina. At least for the Cyp2c44 isoform that was previously attributed a pro-angiogenic action in *in vitro* studies and tumors (Yang et al., 2009; Pozzi et al., 2010), it seems that in the murine retina the enzyme is restricted to Müller glia cells (Hu et al., 2017b). Interestingly, in the retina the sEH is also absent from endothelial cells but expressed in Müller glia cells as well as to a smaller extent in retinal astrocytes (Hu et al., 2014).

One CYP enzyme that may be expressed in retinal endothelial cells is Cyp1b1. However, Cyp1b1 seems to be a somewhat enigmatic enzyme and although it was regularly reported to be the mRNA most upregulated following the application of fluid shear stress to cultured endothelial cells (Garcia-Cardeña et al., 2001; Dekker et al., 2002; Conway et al., 2009), it proved impossible to detect the protein, despite the fact that the available antibodies have no problem detecting the recombinant or overexpressed protein. This enzyme is worth mentioning here as its deletion impaired revascularization in a model of oxygen-induced retinopathy in mice (Tang et al., 2009). Although Cyp1b1 is an estrogen metabolizing CYP hydroxylase, the latter effects in the eye were linked with a decrease in the expression of the endothelial nitric oxide synthase (Tang et al., 2010), as well as a corresponding increase in intracellular oxidative stress and increased production of thrombospondin-2, an endogenous inhibitor of angiogenesis (Tang et al., 2009; Palenski et al., 2013). It has been speculated that CYP1B1 may mediate the effects of estrogen-induced angiogenesis which is also linked with to changes in endothelial nitric oxide synthase, thrombospondin, and free radical generation (Tang et al., 2009; Palenski et al., 2013). Certainly, the CYP1B1-derived metabolites of β-estradiol promote angiogenesis in uterine artery endothelial cells (Jobe et al., 2010). Other reports, however, suggest the opposite as the induction of Cyp1b1 by indole-3-carbinol is reported to inhibit angiogenesis induced by adipocyte conditioned medium (Wang et al., 2016). More recently, an expression analysis approach applied to β-catenin-deficient endothelial cells identified Cyp1b1 as a β-catenin-regulated gene that affects endothelial cell barrier function (Ziegler et al., 2016), but the same study again failed to convincingly show a change in protein expression. Although Cyp1b1 is probably best known for generating retinoic acid from retinol it was found to generate 20-HETE from arachidonic acid to decrease endothelial barrier function *in vitro*. In mice, the pharmacological inhibition of Cyp1b1 increased the permeability of the blood brain barrier for small molecular tracers. Other studies assessing Cyp1b1 expression in the brain by means of histochemistry detected the protein in smooth muscle cells of some small arteries/arterioles but not in endothelial cells (Granberg et al., 2003).

### Astrocytes

Retinal angiogenesis is closely linked to the underlying astrocyte scaffold (Dorrell et al., 2010), and astrocytes express both CYP and sEH enzymes (Iliff et al., 2009). Indeed, the first evidence of a role for EETs in angiogenesis was obtained in a co-culture model in which the astrocytes generated and released angiogenic PUFA epoxides (Munzenmaier and Harder, 2000; Zhang and Harder, 2002). Somewhat surprisingly, however, although there was a clear defect in retinal endothelial cell proliferation and the branching of the nascent vascular plexus in animals globally lacking the sEH, the astrocyte-specific deletion of the enzyme was without effect on retinal angiogenesis (Hu et al., 2014). Recently Cyp1b1 expression in retinal astrocytes was also reported to contribute to retinal neurovascular homeostasis. Indeed, retinal astrocytes from Cyp1b1^−/−^ mice were more proliferative and migratory, produced more fibronectin, and expressed higher levels of αvβ3 and α5β1 integrins, than cells from wild-type mice (Falero-Perez et al., 2019). These results were consistent with the increased adhesive properties of Cyp1b1-deficient astrocytes and their lack of ability to form a network on Matrigel, hinting that Cyp1b1 deletion led to increased proliferation and diminished oxidative stress (Falero-Perez et al., 2019). Even through Cyp1b1 expression was able to rescue the phenotype of Cyp1b1-deficient cells, changes in Cyp1b1 protein expression were not demonstrated.

### Monocytes/Macrophages

Not a lot is known about the control and consequences of CYP-sEH pathway activation in monocytes and macrophages but macrophages do express the sEH as well as a number of different CYP enzymes including CYP2J2, CYP2C8 (Nakayama et al., 2008; Bystrom et al., 2011), and CYP2S1 (Frömel et al., 2013; Behmoaras et al., 2015). Although these enzymes generate EETs and monocytes also express high affinity binding sites for EETs (Wong et al., 2000), there has been no detailed investigation of EET-signaling in these cells. However, given that CYP enzymes also generate superoxide anions (Fleming et al., 2001b), CYP activation may also contribute to radical production and inflammatory activation. There is, however, a link between the Tie2-driven overexpression of the human CYP2C8 isoform and pathologic retinal neovascularization in mice. Although not definitively shown, it was suggested that the CYP2C8 expressed in a subset of Tie-2-expressing monocytes/macrophages was upregulated during oxygen-induced retinopathy, while, fitting with its regulation by hypoxia (Keserü et al., 2010), sEH expression was suppressed to result in an increased retinal epoxide:diol ratio (Shao et al., 2014). Interestingly, when animals were given a ω-3 PUFA-rich diet retinal neovascularization increased, while there was no effect of the diet in animals maintained under normoxic conditions. A second CYP enzyme of interest is CYP46A1, which converts cholesterol to 24-hydroxycholesterol, as retinas from Cyp46a1^−/−^ mice exhibit venous beading and tortuosity, microglia/macrophage activation, and increased vascular permeability, features commonly associated with diabetic retinopathy (Saadane et al., 2019). Mechanistically, the effects were linked to the ability of the product, i.e., 24-hydroxycholesterol to act as a ligand of the liver X receptor (LXR). Indeed, the expression of Lxrα and Lxrβ were increased in the Cyp46a1^−/−^ retina as well as in isolated retinal microglia/macrophages. Retinal endothelial cells also expressed the enzyme and its expression was increased in the pro-inflammatory environment. Comparison of the retinal phosphoproteomes revealed that Cyp46a1-deficiency altered the phosphorylation of 30 different proteins, including tight junction protein zonula occludens 1 and aquaporin 4 (Saadane et al., 2019), although it is not entirely clear how many of these effects can be linked with LXR. CYP2S1 is interesting as it was identified in two studies, one using a proteomic approach (Frömel et al., 2013), and the other using high throughput RNA sequencing (Behmoaras et al., 2015) and is reported to be the CYP enzyme most highly expressed in human monocytes. Its function is unusual in that it metabolizes prostaglandin (PG) G_2_ and PGH_2_ to 12(S)-hydroxyheptadeca-5Z,8E,10E-trienoic acid, thus preventing the generation of other PGs. This is relevant inasmuch as a decrease in PGE_2_ production would certainly be expected to result in a macrophage subtype with attenuated angiogenic potential (Frömel et al., 2013). Interestingly, while CYP2S1 is expressed in classically activated macrophages, it is not expressed in tumor associated macrophages but it is not yet clear whether the differential expression of the enzyme is a cause or consequence of macrophage polarization (Frömel et al., 2013).

### Müller Cells

In the human and murine retina the expression of the sEH is concentrated in retinal Müller glia cells (Hu et al., 2014; Hu et al., 2017a), in mice the same is also the case for Cyp2c44 (Hu et al., 2017b). This is of relevance as Müller glia cells develop and maintain a close contact with both superficial vessels and deeper capillaries *via* their multiple end feet (Newman and Reichenbach, 1996), and these cells have been implicated in angiogenesis by virtue of their ability to produce angiogenic substances in response to hypoxia (Stone et al., 1995; Pierce et al., 1995; Robbins et al., 1997). For a long time Müller cells were assumed to play a greater role in proliferative retinopathy than physiological angiogenesis, as the Müller cell-specific deletion of VEGF-A inhibited neovascularization in a mouse model of oxygen-induced retinopathy without affecting physiological vascularization or retinal morphology (Bai et al., 2009; Hu et al., 2014). It is now clear that while VEGF plays a major role in angiogenesis Müller cells can contribute to retinal angiogenesis *via* other signaling mediators. One example, is Norrin, a retinal signaling molecule secreted by Müller cells that binds to Frizzled-4 to activate canonical Wnt/β-catenin signaling: without Norrin the development of the superficial retinal vessels was attenuated and deeper intraretinal capillaries failed to form (Xu et al., 2004; Ye et al., 2009; Ohlmann and Tamm, 2012). The developing retinal vasculature is exposed to a hypoxic microenvironment and the deletion of HIF-1α in neuroretinal cells (includes Müller cells) resulted in impaired vascular development characterized by decreased tip cell filopodia and reduced vessel branching (Nakamura-Ishizu et al., 2012; Hu et al., 2014). The latter phenotype is very similar to that reported in sEH^−/−^ mice, and the deletion of the sEH in Müller cells inhibited endothelial cell proliferation as well as Notch signaling and tip cell filopodia formation, indicating that Müller cell PUFA metabolites make an important contribution to retinal angiogenesis (Hu et al., 2014). Theoretically, the phenotype associated with sEH deletion could have been attributed to the accumulation of a substrate epoxide or the lack of a product diol. In the case of retinal angiogenesis, the phenotype was attributed to the lack of the DHA-derived diol; 19,20-DHDP.

Once Cyp2c44 was identified in Müller glia cells it was assumed that this enzyme delivered the DHA-derived epoxides for further metabolism by the sEH. Indeed, given that the vascular defects observed in retinas from sEH^−/−^ mice were attributed to Notch activation (Hu et al., 2014), it was expected that the phenotype observed in retinas from Cyp2c44^−/−^ mice would be associated with Notch inhibition. Certainly, the constitutive as well as inducible postnatal genetic deletion of Cyp2c44 resulted in an increased vessel network density without affecting vessel radial expansion during the first postnatal week and was concomitant with the down-regulation of molecules involved in the Notch signaling pathway (Hu et al., 2017b). However, while 19,20-DHDP could be implicated in the defective angiogenesis in sEH deficient mice it was not possible to link the retinal phenotype in 5 day old Cyp2c44^−/−^ mice with a distinct change in the ω-3 and ω-6 PUFA metabolite profile. Despite the fact that in *in vitro* studies Cyp2c44 was able to metabolize arachidonic acid, linoleic acid, EPA and DHA, metabolites from none of these PUFAs were altered in Cyp2c44-deficient retinas (Hu et al., 2017b). A lack of effect of Cyp2c44 deletion on PUFA epoxide and diol levels is consistent with observations made while assessing the role of Cyp2c44 in the lung and heart (Joshi et al., 2016). Rather the consequences of Cyp2c44 deletion were attributed to elevated aldosterone and “as-yet-unknown systemic factors” rather than to altered epoxide generation (Joshi et al., 2016). In the Cyp2c44-deficient murine retina, hydroxydocosahexaenoic acids (HDHA), i.e., 10-, 17-, and 20-HDHA were found to be significantly elevated and were identified as potential Cyp2c44 substrates (Hu et al., 2017b). HDHAs are of interest given that they can be further metabolized to produce a series of specialized “pro-resolving” lipid mediators termed the protectins, resolvins, and maresins (Serhan, 2014). Importantly, the HDHA metabolite 17-oxo-DHA has been attributed anti-inflammatory properties linked to the transcription factor Nrf2 (Cipollina et al., 2014; Gruber et al., 2015), which has in turn been linked with Notch activation; for review see Wakabayashi et al. (2015). Whether or not the increased levels of HDHA in retinas from Cyp2c44^−/−^ can be linked to Notch signaling *via* Nrf2 remains to be determined. An alternative explanation could be the generation of an alternative angiogenic mediator not generally included in targeted mass spectrometry-based screens, possible examples being 17S-hydroxy-containing docosanoids and 17S series resolvins that are reportedly biosynthesized *via* epoxide-containing intermediates in murine brain, human blood, and glial cells (Hong et al., 2003; Hu et al., 2017b).

## Retinal Vascular Pathology

Neovascular eye diseases, including retinopathy of prematurity, diabetic retinopathy, and age-related macular degeneration, threaten vision, and impair quality-of-life. Currently available treatment options, such as anti-VEGF therapy and laser ablation, have limitations and side effects, thus alternative options are required. Clinical and experimental studies indicate that dietary ω-3 PUFAs can affect retinal and choroidal angiogenesis. For example, the ω-3 PUFA metabolites generated by cyclooxygenases and lipoxygenases, inhibit inflammation and angiogenesis, while the ω-6 PUFA metabolites do the opposite (Gong et al., 2017). Given that ω-3 and ω-6 PUFA products of CYP2C enzymes were found to promote neovascularization in the retina and choroid, it was suggested that CYP inhibition might prove beneficial (Gong et al., 2016a). However, given the known side effects of CYP inhibition and novel data linking ω-3 PUFA diols with deleterious effects a therapy directed against the sEH may prove more effective.

### Retinopathy of Prematurity

Retinopathy of prematurity is a complication of treating preterm infants with underdeveloped lungs with high concentrations O_2_. It is estimated that as many as 10% of very premature infants become blind as a consequence of aberrant retinal neovascularization that leads to fibrovascular retinal detachment (Connor et al., 2009; Rivera et al., 2017). Treatment strategies have focused on vascular ablative therapy and more recently on anti-VEGF-based approaches, but these strategies come with adverse side effects and cannot prevent the recurrence of the disease (Chan-Ling et al., 2018; Sternberg and Durrani, 2018). Another potential contributor to the pathology is a deficiency of ω-3 PUFAs, particularly DHA (Lapillonne and Moltu, 2016; Rivera et al., 2017). Although dietary supplementation has shown some promise in preventing retinopathy of prematurity (Rivera et al., 2011; Pawlik et al., 2014), and has been linked with a coincident normalization of circulating adiponectin levels by modulating endoplasmic reticulum stress in white adipose tissue (Fu et al., 2015), exactly how the beneficial effects in the retina are achieved is unclear. However, given the link between the ω-3 PUFA sEH product; 19,20-DHDP, and diabetic retinopathy (Hu et al., 2017a), infants with higher sEH expression are less likely to benefit from the supplementation with ω-3 PUFAs. An alternative approach would be to prevent the generation of the sEH substrate and target the CYP enzymes that are responsible for epoxide production. Indeed, in a mouse model of retinopathy of prematurity the inhibition of CYP2C enzymes was reported to potentiate the protective effects of ω-3 PUFA on retinal neovascularization and choroidal neovascularization (Gong et al., 2016a). In CYP2C8-overexpressing mice fed a ω-3 PUFA diet, CYP inhibition suppressed retinal neovascularization and choroidal neovascularization while sEH inhibition increased oxygen-induced retinopathy and choroidal neovascularization (Gong et al., 2016a).

### Macular Degeneration

Age-related macular degeneration (AMD) is linked to the abnormal growth of choroidal blood vessels and neovascularization is a hallmark of the neovascular (wet) form of advanced AMD. A potential role for PUFA metabolites in AMD has been speculated on the basis of the observation that dietary supplementation with ω-3 PUFAs promoted the regression of choroidal neovessels in a mouse model of AMD (Yanai et al., 2014). The serum of mice given the dietary supplement showed increased levels of 17,18-EEQ and 19,20-EDP, the major CYP-generated metabolites of EPA and DHA. Supplementation also decreased inflammation i.e. leukocyte recruitment and adhesion molecule expression in choroidal neovascular lesions, leading to the conclusion that CYP-derived ω-3 PUFA metabolites are potent inhibitors of intraocular neovascular disease (Yanai et al., 2014). Fitting with this the epoxides of EPA and DHA were found play a significant role in dampening the severity of laser-induced choroidal neovascularization in the mouse (Hasegawa et al., 2017). In the latter study either the overexpression of CYP2C8 or the deletion/inhibition of the sEH resulted in an increase in EDP and EEQ levels as well as in attenuated choroidal neovascularization. The opposite approach i.e. the overexpression of the sEH resulted in the loss of the protective effect. While these findings suggest that the beneficial effects of dietary supplementation were attributable to the anti-inflammatory effects of the ω-3 PUFA epoxides, it may also be the case that the decreased generation of a pro-inflammatory mediator; such as a ω-3 PUFA diol, could explain the observations made. At this point it is important to note that a significant increase in the retinal expression of the sEH has been reported in human eyes (obtained postmortem) from subjects with wet AMD compared to age-matched controls (Sulaiman et al., 2018). The latter observation would rather imply that an sEH product such as a ω-3 PUFA diol, could contribute to the pathogenesis of the disease. Thus, there seems to be a yin and yang relationship between the actions of the ω-3 PUFA epoxides and diols in the retina, at least at the phenotypic level.

### Diabetic Retinopathy

Diabetic retinopathy is an important cause of blindness in the adult population (Yau et al., 2012; Bourne et al., 2013), and is characterized by an initial stage (non-proliferative retinopathy) characterized by the progressive loss of vascular cells and the slow dissolution of inter-endothelial tight junctions resulting in vascular leak and retinal edema (Das et al., 2015). Later stages of the disease are characterized by inflammatory cell infiltration, tissue destruction, and neovascularization (Robinson et al., 2012; Klaassen et al., 2013). Given that the early initiating event(s) of the disease are unknown no effective treatment exists that can be applied to effectively stop or delay degeneration prior to the development of hypoxia and the upregulation of VEGF that signals the progression to proliferative retinopathy.

Almost a decade ago, the first evidence that the CYP-sEH pathway was activated in diabetic retinopathy was obtained by analyzing the vitreous recovered from individuals undergoing vitreoretinal surgery. The samples studied revealed a diabetes associated increase in 5-HETE and EETs as well as a number of unknown PUFA metabolites (Schwartzman et al., 2010). Analyses of differentially expressed retinal genes linked to streptozotocin-induced diabetic retinopathy in rats identified eight candidates that were differentially expressed at different time points; the latter included the downregulation of Cyp2b2 after 1 week (Zhao et al., 2017). This however, contrasts with reports that hypoxia (which occurs in retinopathy) increases CYP2C expression in retinal endothelial cells (Michaelis et al., 2008), and that fenofibrate; which binds to and inhibits CYP2C, reduced retinal and choroidal neovascularization in PPARα^−/−^ mice and augmented ω-3 PUFA protection *via* CYP2C inhibition (Gong et al., 2016b). Although CYP expression could be expected to increase as a result of the hypoxia experienced in the retina in the later stages of the disease, the retinopathy induced by streptozotocin tends to be milder than that observed in genetic models of diabetes and rarely reaches the proliferative stage. However, in a Japanese population CYP2C19 loss of function polymorphisms have been associated with an increased risk of diabetic retinopathy, albeit only in female patients (Kajiwara et al., 2013).

Recently, the DHA-derived diol; 19,20-DHDP, was implicated in the development of diabetic retinopathy and attributed to a pronounced increase in the expression of the sEH (Hu et al., 2017a). Not only was 19,20-DHDP elevated in mice with a genetic form of diabetes but the metabolites was also detected in the vitreous humor from diabetic human subjects. High concentrations of 19,20-DHDP are detrimental to vascular integrity and barrier function as it interacts with cholesterol in the cell membrane to alter the localization of cholesterol-binding proteins. As such 19,20-DHDP interfered with the association of PS-1 with N-cadherin and VE-cadherin to compromise pericyte-endothelial cell as well as inter-endothelial cell junctions and promote the dissolution of the blood-retinal barrier (Hu et al., 2017a) (**Figure 3B**). Not only did the overexpression of the sEH in healthy non-diabetic mice induce a retinopathy very similar to that of non-proliferative diabetic retinopathy but the treatment of diabetic mice with an sEH inhibitor prevented the pericyte loss and vascular permeability that characterize diabetic retinopathy (Hu et al., 2017a). The molecular events leading to the increase in sEH in diabetes are not known but one interesting possibility is *via* the histone demethylase Jarid1b, that was recently reported to control the 3’ untranslated region of the sEH (Vasconez et al., 2019).

While much of the evidence linking sEH with a particular eye disease was gained from targeted studies, i.e. they started out from the knowledge/assumption that sEH expression or activity could play a major role in the retina, a recent untargeted approach led to the same conclusion. The study in question did not so much target the sEH but rather started out with a pharmacologically effective compound and screened for its target. More specifically, affinity reagents based on a homoisoflavonoid derivative, SH-11037, that was reported to significantly attenuate angiogenesis in the laser-induced choroidal neovascularization model in the mouse (Grossniklaus et al., 2010), was used as a target in a proteomic approach. The SH-11037-based reagents were immobilized and used to pull down protein binding partners from a porcine brain lysate, resulting in the recovery of the sEH (Sulaiman et al., 2018). It turned out that the compound bound to the catalytic site of the sEH, to inhibit its activity. Even though SH-11037 was less efficient than some of the sEH inhibitors used on other animal studies, the compound inhibited the sEH *in vitro via* a novel interaction and partially normalized the 19,20-EDP/-DHDP ratio after the induction of neovascularization in mice (Sulaiman et al., 2018).

The latter studies focused on the early non-proliferative form of diabetic retinopathy, but as PUFA epoxides have been implicated in angiogenesis, is there any evidence that they contribute to the later stages of the disease usually characterized by retinal hypoxia by an increase in VEGF production? This would be an attractive hypothesis given that VEGF increases the activity of the CYP2C promoter to enhance CYP2C expression and activity and increase intracellular EET levels in endothelial cells (see section CYP-Derived PUFA Mediators and Angiogenesis). While the epoxides of arachidonic acid promote angiogenesis, the CYP2C-derived epoxides of EPA seem to inhibit it, and 17,18-epoxyeicosatetraenoic acid, which is derived from EPA can activate the growth-suppressing p38 MAP kinase and downregulate cyclin D1 to inhibit cell proliferation in an immortalized endothelial cell line (Cui et al., 2011). Less is known about the biological actions of the DHA-derived epoxides but EDPs have been reported to inhibit inflammation in human retinal microvascular endothelial cells (Capozzi et al., 2016) as well as in a mouse model of choroidal neovascularization (Hu et al., 2017a), and have been linked with pathological neovascularization (Shao et al., 2014; Gong et al., 2016a). Indeed, the induction of retinopathy of prematurity in mice with a Tie2-driven overexpression of human CYP2C8 and fed with an ω-3 PUFA-rich diet clearly increased angiogenesis, an effect that correlated with increased plasma levels of 19,20-EDP as well as increased retinal VEGFA mRNA expression (Shao et al., 2014). Moreover, the inhibition of CYP2C to reduce EDP levels, suppressed neovascularization in the mouse model of retinopathy of prematurity as well as in a model of choroid injury. Inhibition of the sEH to prevent the metabolism of EDP in the retina, on the other hand, resulted in increased neovascularization (Gong et al., 2016a).

## Outlook

Retinal vascular diseases have devastating impact on the quality of life and although ablation and anti-VEGF therapies can be used to manage the symptoms of the later stages of retinopathy there is a clear need for therapies that can effectively delay disease development. At least for AMD and diabetic retinopathy, inhibition of the sEH may be an interesting option to target metabolites that are actively involved in disease pathogenesis.

## Author Contributions

IF wrote the manuscript.

## Conflict of Interest Statement

The author declares that the research was conducted in the absence of any commercial or financial relationships that could be construed as a potential conflict of interest.

## Abbreviations

Adult respiratory stress syndrome (ARDS); age-related macular degeneration (AMD); Ca^2+^-activated potassium channels (K_ca_ channels); cytochrome P450 (CYP); dihydroxydocosapentaenoic acid (DHDP); dihydroxyeicosatrienoic acid (DHET); dihydroxyoctadecenoic acid (DiHOME); docosahexaenoic acid (DHA); epoxydocosapentaenoic acid (EDP); eicosapentaenoic acid (EPA); epoxyeicosatrienoic acid (EET); epoxyoctadecenoic acid (EPOME); G protein coupled receptor (GPR); hydroxyeicosatetraenoic acids (HETE); hypoxia inducible factor (HIF); notch intracellular domain (NICD); peroxisome proliferator-activated receptor (PPAR); polyunsaturated fatty acid (PUFA); potent liver X receptor (LXR); presenilin 1 (PS-1); prostaglandin (PG); protein kinase (PK); soluble epoxide hydrolase (sEH); transient receptor potential channel (TRP channel); vascular endothelial growth factor (VEGF).
